# Interplay between Abscisic Acid and Gibberellins, as Related to Ethylene and Sugars, in Regulating Maturation of Non-Climacteric Fruit

**DOI:** 10.3390/ijms22020669

**Published:** 2021-01-12

**Authors:** Fernando Alferez, Deived Uilian de Carvalho, Daniel Boakye

**Affiliations:** 1 Southwest Florida Research and Education Center, Department of Horticulture, University of Florida–Institute of Food and Agricultural Sciences (UF–IFAS), Immokalee, FL 34142, USA; deived10@gmail.com (D.U.d.C.); dadu.boakye@ufl.edu (D.B.); 2 AC Jardim Bandeirante, Centro de Ciências Agrárias, Universidade Estadual de Londrina, Jardim Portal de Versalhes 1 86057970, Londrina/PR 10011, Brazil

**Keywords:** abscisic acid, citrus, ethylene, fruit maturation, gibberellins, hormonal interplay, sugars

## Abstract

In this review, we address the interaction between abscisic acid (ABA) and gibberellins (GAs) in regulating non-climacteric fruit development and maturation at the molecular level. We review the interplay of both plant growth regulators in regulating these processes in several fruit of economic importance such as grape berries, strawberry, and citrus, and show how understanding this interaction has resulted in useful agronomic management techniques. We then relate the interplay of both hormones with ethylene and other endogenous factors, such as sugar signaling. We finally review the growing knowledge related to abscisic acid, gibberellins, and the genus Citrus. We illustrate why this woody genus can be considered as an emerging model plant for understanding hormonal circuits in regulating different processes, as most of the finest work on this matter in recent years has been performed by using different Citrus species.

## 1. Introduction

The development and maturation of fruit is the result of a complex interplay of molecular, biochemical, and physiological processes, modulated by internal factors such as hormones and external factors such as the environment. In general, the transition from fruit growth to maturation involves changes in the sugar metabolism, and the softening and coloration of different fruit tissues. The development of fleshy fruit is divided into three distinct stages: the first stage (1) is recognized by slow growth as cell division takes place; the second stage (2) is marked by a rapid fruit growth due to cell expansion, and major increases in size and weight are observed; fruit ripening is initiated at the third stage (3), when fruit growth ceases and there is an increase in the biochemical reactions that result in fruit maturation involving fruit color-change, acid degradation, sugar accumulation, and other processes that combined result in final organoleptic attributes. Classically, fleshy fruits are classified into two physiological categories based on the respiration pattern and ethylene biosynthesis occurring at stage 3: climacteric and non-climacteric [1]. Climacteric fruits, such as tomato, apple, apricot, atemoya, banana, blueberry, guava, mango, papaya, and peach have an increase in the respiration rate and ethylene production at stage 3 when fruit ripening process enable fruit harvest prior to complete fruit maturation. On the other hand, non-climacteric fruits including strawberry, citrus, grape, cherry, plum, litchi, and others display a progressive reduction in the respiration rate during maturation while the ethylene production remains at basal level. The hormonal regulation of fruit ripening in climacteric fruit has been widely addressed, and in-depth studies, taking into account molecular aspects of hormone crosstalk and interaction with environment are abundant, greatly thanks to research on tomato, a very well characterized model plant due to a wide collection of mutants available and a faster growth cycle [2,3,4]. Hormonal interaction in regulating maturation of non-climacteric fruit has been also studied in several fruits including strawberry, grape berries and citrus among others. Whereas observational studies are relatively abundant, in-depth molecular and mechanistic studies have been performed only in a handful of fruit including strawberry, grape berries and citrus, mostly because of their economic importance. However, the focus of these kind of studies have been primarily on ethylene and its interaction with other hormones. In this review, we want to focus on interaction between abscisic acid (ABA) and gibberellins (GAs) in regulating non-climacteric fruit development and maturation at the molecular level. We then relate the interplay of both hormones with ethylene and other endogenous factors, such as sugar signaling. We finally review the growing knowledge related to abscisic acid, gibberellins, and the genus Citrus. We highlight how Citrus can be considered as an emerging model plant for understanding hormonal circuits in regulating different physiological processes, including fruit maturation and responses to stress, as most of the finest work on this matter in the last years has been performed by using different Citrus species.

## 2. Introduction to Abscisic Acid in Fruit

### 2.1. Abscisic Acid Biosynthesis and Accumulation

Accumulation of ABA in both climacteric and non-climacteric fruit during maturation is known for decades [5,6,7,8,9]. ABA is a product of the carotenoid pathway [5,6]. This plant hormone is derived from C40-*cis*-epoxycarotenoids, which are cleaved by the 9-*cis*-epoxycarotenoid dioxygenase (NCED) to produce xanthoxin, the direct C15 precursor of ABA [7,8,9]. Several studies in non-climacteric fruit have shown the role of this hormone in regulating the process of maturation. In cherries, endogenous ABA levels are the result of a balance between biosynthesis mediated by *PacNCED*s, and catabolism mediated by *PacCYP707A*s (encoding a 8’-hydroxylase, a key enzyme in the oxidative catabolism of ABA), and transcriptional regulation of these genes influence maturation [10,11]. In mangosteens, ABA accumulation in fruit peel and aril precedes fruit coloration and decrease in peel firmness, suggesting the involvement of this hormone in triggering maturation [12]. In Citrus, increase in ABA concentration in the fruit occurs during maturation in different species [13,14,15] and irrespective of the fruit tissue [16]. Lowering levels of ABA are accompanied by a delay in color change in lemons, and retarding senescence has been related also to lower levels of this hormone [17,18]. In addition, it has been noted a relation between ABA increase and transition from chloroplast to chromoplast in mandarin [19] and sweet cherry [10]. In Citrus, ABA increases in response to ethylene [19,20], and accumulates during fruit development, maturation and senescence [13,17].

### 2.2. Abscisic Acid Function During Fruit Maturation

Exogenous treatments with ABA may also have an effect in different maturation parameters, although there are some disparities in the response depending on the fruit. For instance, in field-grown grape berries, exogenous ABA increases maturation-related pigments such as anthocyanin and flavonol [21,22,23], advances the process of color change (veraison), and downregulates expression of genes associated with photosynthesis [24]. Interestingly, ABA may exert different actions on maturation and on ethylene biosynthesis depending on the stage of fruit development in grape [25].

The involvement of ethylene in non-climacteric maturation is not the focus of this review as there are many in-depth studies of these interactions [26] and will be addressed in Section 4, Integrating Signals to Regulate Maturation: GA, ABA, Sugars, and Ethylene Interaction. However, in the context of this article, it is worth to mention that combined application of ABA and the ethylene releasing compound ethrel to *Litchi chinensis* three weeks before harvest was more effective in enhancing both chlorophyll degradation and anthocyanin biosynthesis than the application of ABA alone, showing a possible synergistic effect of ABA and ethylene in promoting anthocyanin synthesis, chlorophyll degradation and ultimately peel coloration. Interestingly, in this study, exogenous ABA also induced sugar accumulation [27]. Exogenous application of ABA before color break also improved color in mandarin fruit (*Citrus reticulata* Blanco cv. Ponkan) [28] and in M7 sweet orange [29]; however, in other citrus fruit exogenous ABA did not promote color development [30], whereas in juice sacs cultured in vitro, ABA induced its own biosynthesis at the transcriptional level, and this feedback regulation of ABA led to a decrease in carotenoid content [31]. The nature of ABA synthesis, being a final product of the carotenoid biosynthetic pathway, makes particularly difficult to unravel and ultimately understand its role in coloration when carotenoids are the main pigments involved. This elusiveness can be largely avoided in fruit such as grapes, accumulating other classes of pigments, such as anthocyanins, as we discussed above.

## 3. GAs, ABA and Their Interplay during Fruit Development and Maturation

### 3.1. Integration of ABA and GAs Biosynthesis

**Figure 1:**
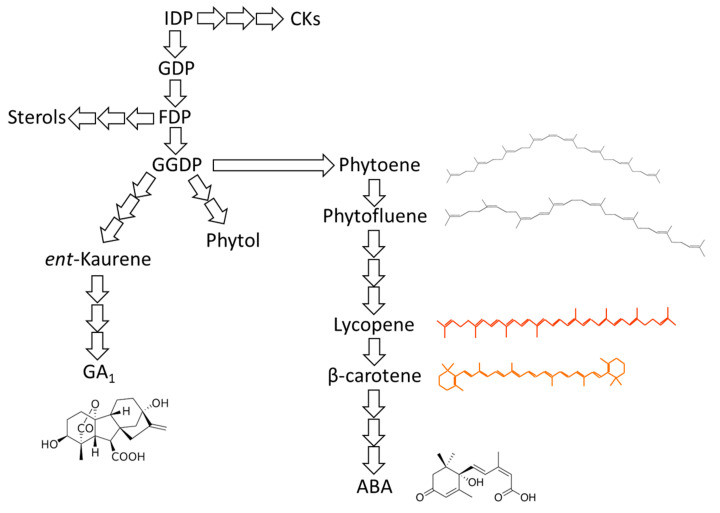
General scheme of pathways leading to production of different hormones. ABA and GAs share their biosynthetic pathway with other hormones, such as CKs. During fruit maturation, balance among hormone biosynthesis changes, and this involves carotenogenesis. IDP, isopentenyl diphosphate; GDP, geranyl diphosphate; FDP, farnesyl diphosphate; GGDP, geranyilgeranyl diphosphate . Number of arrows illustrate number of biosynthetic steps.

ABA and GAs share their biosynthetic pathway with other plant growth regulators, including cytokinin (CK) and diverse sterols (Figure 1). In model plants, several GAs and ABA mutants have been identified and characterized, allowing the elucidation of their biosynthetic pathway and function. For example, *flacca* and *sitiens* mutants of tomato are defective in the last steps of carotenoids biosynthesis, thus impairing ABA synthesis with downstream effects [9]. In corn, studies on several viviparous mutants helped to elucidate the biochemistry of carotenoid biosynthesis; these mutants show blockages at different steps of the pathway, resulting in accumulation of precursors and reduction of ABA content [32,33]. Certainly, in woody plants such as Citrus the use of mutants altered in hormonal biosynthesis is much less feasible as artificially induced mutants are difficult to generate due to cost and lack of facile methods.

The antagonistic effects of both ABA and GAs in regulating different developmental processes and responses to stress are well known; from a biosynthetic point of view, there exists a competition for the metabolic precursor geranylgeranyl pyrophosphate (GGPP) between GA, phytol and carotenoids biosynthetic pathways [34,35]. In the case of GAs, GGPP undergoes cyclization to *ent*-kaurene and then oxidation to GA_12_-aldehyde, the precursor of all GAs [36]. ABA is synthesized through C15 intermediates after oxidative cleavage of some xanthophylls [37]. Gibberellins (GAs) are tetracyclic diterpenoid carboxylic acids and are also involved in fruit growth and maturation [38]. More than a hundred GAs have been identified in vascular plants [39], but only a few are biologically active [26,40,41]. The main bioactive forms of GAs are GA_1_, GA_3_, GA_4_ and GA_7_. These molecules commonly have a hydroxyl group on C-3β, a carboxyl group on C-6, and a lactone between C-4 and C-10 [40].

### 3.2. GA Biosynthesis During Fruit Development and Maturation

Previous studies have reported the occurrence of these bioactive forms of GAs in different species including non-climacteric fruits, and differential biosynthesis during the processes of fruit development and maturation. In grape berries, expansion of the berry fruit induced by GA_3_ may be linked to the upregulation of cellulose synthase A catalytic subunit genes [42]. Exposure to ABA and GA can induce expression of *Vitis vinifera* Hexose Transporters *VvHT2*, *VvHT3* and *VvHT6* in grape berries during the ripening period when sugar unloading from the phloem is favored [43]. Other genes are also expressed at the late stage of grape berry fruit development and ripening, such as the *Vitis vinifera* SBP-box-like18 *(VvSPL18*), that is significantly upregulated by GA at veraison through an ABA-independent pathway and at the late stage of the berry pericarp ripening process, showing its regulation on maturation [44,45].

In strawberry, recent studies have reported the GA association with fruit development and ripening [41,46,47,48]. The enlargement of receptacle cells during fruit development is regulated by endogenous GAs [41]. The overexpression of the Gibberellin Stimulated Transcript 2 (*FaGAST2*) gene in different strawberry transgenic lines promoted a reduction in fruit size [46]. Silencing of *FaGAST2* resulted in increase of *FaGAST1* expression, but no changes in fruit cell size were noted; this suggests an orchestrated role of both genes at the transcriptional level in controlling fruit size [26,46].

Accumulation of GAs and their metabolism are also important factors controlling maturation in non-climacteric fruits. The presence of bioactive GA_1_, GA_3_ and GA_4_ has been reported during strawberry fruit development. GA_1_ and GA_4_ are most abundant at the early stages of fruit development, and decrease as the strawberry fruit ripens [49,50]. The GA_4_ content in strawberry receptacles is higher than that of GA_1_ and GA_3_, which suggests a major role of GA_4_ in the developmental processes underlying the receptacle transition from green to white, and subsequently to red [41]. Expression of genes encoding GA pathway components involved in GA biosynthesis (*FaGA3ox*) and catabolism (*FaGA2ox*) is higher in the receptacle during strawberry fruit development [41]. Expression of *FaGA3ox*, which is involved in biosynthesis of bioactive GA_4_, is maximum at the green stage while the expression level of *FaGA2ox*, which is involved in the inactivation of this active GA, increases during ripening, and peaking at the red stage [40,41]. Moreover, the expression of *FaGA3ox* in green receptacle is 40 times higher than the expression in the green achene, suggesting that this gene has a prominent role in GA signaling in this tissue [41]. Then, considerable decline in the bioactive forms of GAs is observed at the later stage of fruit development following by the expression of the *FaGA2ox* gene that encodes key enzymes of GA inactivation. These observations, taken together, indicate the degradation of active GA and their content reduction during fruit development, and before maturation processes start.

### 3.3. Exogenous GA Affect Fruit Maturation and ABA Levels in Fruit

The effect of exogenous GA delaying fruit maturation is well known, and leverage of this knowledge has resulted in common horticultural management practices. For instance, the exogenous application of GA_3_ has an inhibitory effect on strawberry ripening, which is evidenced by the delay in anthocyanin synthesis and the decrease in respiration, as well as the reduction in phenylalanine ammonia-lyase (PAL), chlorophyllase and peroxidase activities, enzymes involved in chlorophyll metabolism. This results in delay of degreening [51,52]. Similarly, in citrus GA_3_ treatment is commonly adopted as a degreening-delay strategy, aimed at managing harvesting dates, when applied before the onset of color break [53]. It has been shown that GA maintains higher content of lutein and prevents accumulation of downstream phytoene, phytofluene and xanthophylls leading to ABA synthesis [54,55].

As mentioned above, ABA and GAs have antagonistic effects in regulating several processes in plants, and their relative balance differs during fruit maturation. In the peel of Navel oranges, concentration of GA_4_ and GA_1_ declines before color break and this decline precedes the increase in ABA content. Concentration of both GA and ABA follow then an opposite evolution [16,56]. It has been suggested that the decrease in GA concentration and increase of ABA levels in the peel is part of the ripening program that may stimulate other metabolic pathways associated with coloration, including chlorophyll breakdown and pigment accumulation [57].

## 4. Integrating Signals to Regulate Maturation: GA, ABA, Sugars, and Ethylene Interaction

Sugar and ABA signaling are closely related in regulating numerous processes in plants and have been studied in detail in the model plant *Arabidopsis thaliana*. Many of these studies are translatable to crop plants of agronomic interest, Genetic studies have identified several loci involved in both sugar and ABA responses, regulating several developmental processes [58]. Interestingly, many sugar-insensitive *Arabidopsis* mutants are either ABA insensitive (*abi* mutants) or ABA deficient (*aba* mutants). There exist many examples of gene co-regulation between sugars and ABA, and in *Arabidopsis*, 14% of genes upregulated by ABA are induced also in response to glucose [59], whereas several other genes involved in stress responses and carbohydrate metabolism are repressed by both regulators [60]. Additionally, there is an increasing body of evidence connecting ABA and sugar signaling during non-climacteric fruit maturation. For instance, in grape berries, a wealth of data correlates increases in sugar and ABA with the onset of ripening [61,62,63,64], the ripening-related *ASR* gene is induced by sugar and strongly enhanced by ABA [65], and induction of senescence is ABA-independent, whereas deficiency in the hormone seems to accelerate senescence [66]. Interestingly, in this fruit, synthesis of anthocyanins fails if sugar import into the berry is disrupted via phloem girdling prior to the onset of ripening [64]. In addition, applications of both sugars and ABA, as well as management practices that increase ABA content, also increase anthocyanin accumulation [67,68,69,70], and this occurs at the transcriptional level, by induction of gene expression [64]. The genes *VvHT2* and *VvHT6*, that increased expression at veraison after ABA and GA treatment, are the most important sugar transporters across all stages of berry development, with higher expression at the onset of ripening [43]. These authors suggest that both transporters are more related to phloem unloading in sink organs (fruits) than to phloem loading in source organs (leaves). They also emphasize that these transporters may contribute to mobilize a higher content of carbohydrates from leaves to berries, reinforcing the sink strength of fruits at the onset of ripening.

It has been proposed that in fruit from sweet orange, color change during maturation is the consequence of reduction in levels of the active gibberellins GA_1_ and GA_4_, involved in the regulation of sugars and ABA accumulation in the rind [56]. In this sense, girdling, a well stablished crop management practice, results in reduction of carbohydrate content and delayed peel coloration, whereas GA levels do not decline in the fruit, indicating the physiological connection among these signals. This also suggests that decrease in GA concentration in the fruit is part of the maturation program, as the presence of gibberellins prevents fruit color change, and that active GA concentration must diminish in fruit to allow color break, whereas increase in ABA content precedes fruit color development [56].

**Figure 2:**
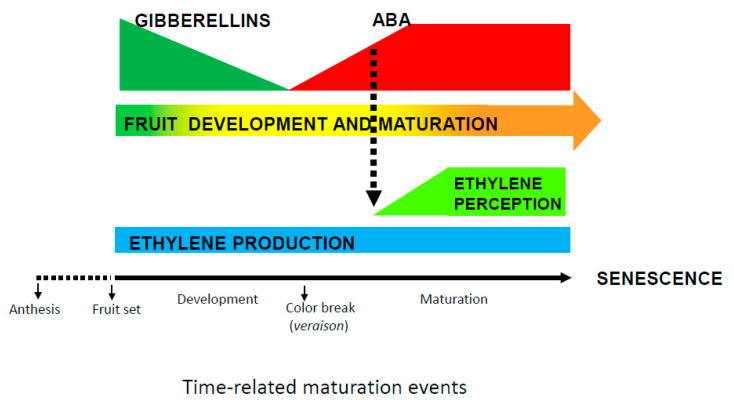
A time-course model of the interplay between abscisic acid (ABA) and gibberellins (GAs) in modulating non-climacteric fruit development and maturation. This is a reductionistic model, as other players involved are not depicted. These include nutritional and environmental factors. The shape of the elements in the figure illustrates the evolution of each component during fruit maturation. GAs decrease, ABA increase and ethylene production remains steady, whereas ethylene perception increases. The crosstalk with ethylene (dash line pointing an induction in ethylene perception driven by ABA) remains to be demonstrated.

Development and maturation of non-climacteric fruit does not require ethylene biosynthesis. However, many of these fruits respond to ethylene during maturation advancing color or increasing size [26], and sensitivity to ethylene could be the key, playing a pivotal role in the process. It has been proposed that changes in the sensitivity to ethylene may be necessary to maintain coloration in the peel of Citrus fruits and that ABA would enhance sensitivity of the fruit to ethylene, as it has been demonstrated in climacteric fruits [71]. Ethylene would then be the stimulator of transcriptional and biochemical changes ultimately associated with maturation [57]. In this scheme, GA levels would concomitantly be reduced as ABA increased (Figure 2).

### Citrus as a Model Plant for Non-Climacteric Maturation Studies

Studies on the role of hormones, their interplay, as well as crosstalk with other factors (i.e., nutritional) in controlling developmental processes and responses to environment have been classically addressed using easy-to-genetically-manipulate model plants. This is the case of *Arabidopsis thaliana*, *Zea mays*, or *Solanum lycopersicum*, in which the availability of mutants impaired in synthesis or perception of a hormone is not a bottleneck for these kind of studies. Although many of the processes studied by using these plant systems can be translated to other agronomically interesting plants, there are specificities, especially in woody plants yielding fleshy fruit that are unique and require more tailored approaches. In many woody plants, usually of agronomic interest, this model plant approach has been traditionally less affordable, due to technical challenges, including in some cases lack of information at the genomic level, unavailability of varieties and/or mutants impaired in hormonal biosynthesis or response, long juvenile period, and difficulties to achieve efficient genetic transformation. Increasingly, this is not the case with Citrus, as in recent years, many species from the genus Citrus have been sequenced, their genealogy revealed, and the sequences made publicly available [72]; Citrus species are prone to spontaneous mutations, with many of these affecting hormonal regulation of maturation, such as ‘Pinalate’, a spontaneous mutant of Navel orange (*Citrus sinensis* L. Osbeck) that presents lower levels of ABA in all fruit tissues as compared to its parental, and ‘Navel negra’, a mutant that is impaired in chlorophyll degradation [5,73]; and finally, genetic transformation has been achieved through diverse engineering techniques, and greatly improved with practical, applicable results [74,75,76]. Together, these advances have helped to elucidate the role of ABA and GAs in the regulation of non-climacteric fruit maturation. The genus Citrus is very diverse, as is comprised by various species and varieties including oranges, mandarins, lemons, grapefruits, pummelos, citrons, limes, kumquats; in addition, different hybrids and spontaneous mutants that have been selected for commercial reasons and are predominantly grown in the tropical and subtropical regions. Citrus develop spontaneous mutations with remarkable frequency in the field. As a result, many of the cultivars currently grown around the world have been obtained by selection of these naturally occurring mutants [5]. Some of these available mutants provide useful aids to dissect some of the processes affected by the mutation [73,77,78]. For instance, the peel of Citrus constitutes an excellent system to investigate the regulation of ABA biosynthesis, signaling and interplay with other hormones and stress regulators during peel maturation [16,79,80,81]. Recently, it has been completed the identification of ABA signaling core components in Citrus, and their function during maturation has started to be unveiled. This complex is comprised of six PYR/PYL/RCAR ABA receptors, five PP2CAs, and two subclass III SnRK2s. During sweet orange fruit development and ripening, the expression pattern of some ABA receptors mirrors the ABA content, whereas that of *CsPP2CA* genes parallels the hormone accumulation, together modulating ABA perception, downstream signaling, and, consequently, physiological ABA responses [77]. Not only have citrus been useful in understanding fruit maturation though the use of mutants, the response of citrus fruit to different stresses has also started to be elucidated using available mutants defective in ABA, as hormonal signaling in response to stress is also modulated, and varies during maturation [78,81]. This has implications in understanding hormonal regulation of the response to postharvest stress and paves the path to better management practices. In any case, to consider Citrus as a model, the knowledge accrued on these studies should be translatable to other genus. 

## 5. Conclusions

In a nutshell, many studies have been done in the last two decades focusing on the integration of hormonal and nutritional signals during non-climacteric fruit maturation, that has pointed at the interplay between ABA and GAs as a major factor controlling the process. However, the fine details of this regulation are still not well understood and some reports show conflicting results as we have mentioned previously. For instance, how ABA levels may determine tissue sensitivity to ethylene and trigger downstream effects, and how GA and other factors including nutritional and environmental cues, interact in the process, is not completely understood. This warrants future research on how sensitivity to ethylene is triggered and regulated, the involvement of sugars and climate in the whole process, if and how downstream processes depend also on this hormonal setup, if these responses are conserved or species-specific, and—from a practical and commercial standpoint—the implications of this phenomenon during postharvest, as they relate and may determine fruit quality.

## References

[1] Cao M., Zheng J., Zhao Y., Zhang Z., Zheng Z.-L. (2018). Network Analysis of Differentially Expressed Genes across Four Sweet Orange Varieties Reveals a Conserved Role of Gibberellin and Ethylene Responses and Transcriptional Regulation in Expanding Citrus Fruits. Trop. Plant Biol..

[2] Klee H.J., Giovannoni J.J. (2011). Genetics and Control of Tomato Fruit Ripening and Quality Attributes. Annu. Rev. Genet..

[3] Seymour G.B., Østergaard L., Chapman N.H., Knapp S., Martin C. (2013). Fruit Development and Ripening. Annu. Rev. Plant Biol..

[4] Kumar R., Khurana A., Sharma A.K. (2013). Role of plant hormones and their interplay in development and ripening of fleshy fruits. J. Exp. Bot..

[5] Rodrigo M.J., Marcos J.F., Alférez F., Mallent M.D., Zacarías L. (2003). Characterization of Pinalate, a novel *Citrus sinensis* mutant with a fruit-specific alteration that results in yellow pigmentation and decreased ABA content. J. Exp. Bot..

[6] Fraser P.D., Bramley P. (2004). The biosynthesis and nutritional uses of carotenoids. Prog. Lipid Res..

[7] Cutler A.J., Krochko J.E. (1999). Formation and breakdown of ABA. Trends Plant Sci..

[8] Liotenberg S., North H., Marion-Poll A. (1999). Molecular biology and regulation of abscisic acid biosynthesis in plants. Plant Physiol. Biochem..

[9] Taylor I.B., Burbidge A., Thompson A.J. (2000). Control of abscisic acid synthesis. J. Exp. Bot..

[10] Kondo S., Gemma H. (1993). Relationship between Abscisic Acid (ABA) Content and Maturation of the Sweet Cherry. J. Jpn. Soc. Hortic. Sci..

[11] Ren J., Sun L., Wu J., Zhao S., Wang C., Wang Y., Ji K., Leng P. (2010). Cloning and expression analysis of cDNAs for ABA 8′-hydroxylase during sweet cherry fruit maturation and under stress conditions. J. Plant Physiol..

[12] Kondo S., Ponrod W., Kanlayanarat S., Hirai N. (2002). Abscisic Acid Metabolism during Fruit Development and Maturation of Mangosteens. J. Am. Soc. Hortic. Sci..

[13] Goldschmidt E.E., Goren R., Even-Chen Z., Bittner S. (1973). Increase in Free and Bound Abscisic Acid during Natural and Ethylene-induced Senescence of Citrus Fruit Peel. Plant Physiol..

[14] Harris M.J., Dugger W.M. (1986). The Occurrence of Abscisic Acid and Abscisyl-β-d-Glucopyranoside in Developing and Mature Citrus Fruit as Determined by Enzyme Immunoassay. Plant Physiol..

[15] Romero P., Lafuente M.T., Rodrigo M.J. (2012). The Citrus ABA signalosome: Identification and transcriptional regulation during sweet orange fruit ripening and leaf dehydration. J. Exp. Bot..

[16] Alférez F. (2001). Regulación Hormonal de la Maduración en Frutos Cítricos y Su relación Con Alteraciones Fisiológicas Durante la Postcosecha. Ph.D. Thesis.

[17] Aung L.H., Houck L.G., Norman S.M. (1991). The Abscisic Acid Content of Citrus with Special Reference to Lemon. J. Exp. Bot..

[18] Valero D., Martínez-Romero D., Serrano M., Riquelme F. (1998). Influence of Postharvest Treatment with Putrescine and Calcium on Endogenous Polyamines, Firmness, and Abscisic Acid in Lemon (*Citrus lemon* L. Burm Cv. Verna). J. Agric. Food Chem..

[19] Lafuente M.T., Martinez-Tellez M.A., Zacarias L. (1997). Abscisic acid in the response of Fortune mandarin to chilling. Effects of maturity and high temperature conditioning. J. Sci. Food Agric..

[20] Brisker H.E., Goldschmidt E.E., Goren R. (1976). Ethylene-induced Formation of ABA in Citrus Peel as Related to Chloroplast Transformations. Plant Physiol..

[21] Peppi M.C., Fidelibus M., Dokoozlian N., Walker M.A. (2006). Abscisic acid applications improve the color of crimson seedless table grapes. Am. J. Enol. Vitic..

[22] Peppi M.C., Walker M.A., Fidelibus M.W. (2008). Application of abscisic acid rapidly upregulated UFGT gene expression and improved color of grape berries. Vitis.

[23] Wheeler S., Loveys B., Ford C., Davies C. (2009). The relationship between the expression of abscisic acid biosynthesis genes, accumulation of abscisic acid and the promotion of *Vitis vinifera* L. berry ripening by abscisic acid. Aust. J. Grape Wine Res..

[24] Koyama K., Sadamatsu K., Goto-Yamamoto N. (2009). Abscisic acid stimulated ripening and gene expression in berry skins of the Cabernet Sauvignon grape. Funct. Integr. Genom..

[25] Sun L., Zhang M., Ren J., Qi J., Zhang G., Leng P. (2010). Reciprocity between abscisic acid and ethylene at the onset of berry ripening and after harvest. BMC Plant Biol..

[26] Fuentes L., Figueroa C.R., Valdenegro M. (2019). Recent Advances in Hormonal Regulation and Cross-Talk during Non-Climacteric Fruit Development and Ripening. Horticulturae.

[27] Wang H., Huang H., Huang X. (2007). Differential effects of abscisic acid and ethylene on the fruit maturation of Litchi chinensis Sonn. Plant Growth Regul..

[28] Rehman M., Singh Z., Khurshid T. (2018). Pre-harvest spray application of abscisic acid (S-ABA) regulates fruit colour development and quality in early maturing M7 Navel orange. Sci. Hortic..

[29] Wang X., Yin W., Wu J., Chai L., Yi H. (2016). Effects of exogenous abscisic acid on the expression of citrus fruit ripening-related genes and fruit ripening. Sci. Hortic..

[30] Iglesias D.J., Cercós M., Colmenero-Flores J.M., Naranjo M.A., Ríos G., Carrera E., Ruiz-Rivero O., Lliso I., Morillon R., Tadeo F.R. (2007). Physiology of citrus fruiting. Braz. J. Plant Physiol..

[31] Zhang L., Ma G., Kato M., Yamawaki K., Takagi T., Kiriiwa Y., Ikoma Y., Matsumoto H., Yoshioka T., Nesumi H. (2011). Regulation of carotenoid accumulation and the expression of carotenoid metabolic genes in citrus juice sacs in vitro. J. Exp. Bot..

[32] Neill S.J., Horgan R., Parry A.D. (1986). The carotenoid and abscisic acid content of viviparous kernels and seedlings of *Zea mays* L.. Planta.

[33] Paiva R., Kriz A. (1994). Effect of abscisic acid on embryo-specific gene expression during normal and precocious germination in normal and viviparous maize (*Zea mays*) embryos. Planta.

[34] Fray R.G., Grierson D. (1993). Identification and genetic analysis of normal and mutant phytoene synthase genes of tomato by sequencing, complementation and co-suppression. Plant Mol. Biol..

[35] Fray R.G., Wallace A., Fraser P., Valero D., Hedden P., Bramley P.M., Grierson D. (1995). Constitutive expression of a fruit phytoene synthase gene in transgenic tomatoes causes dwarfism by redirecting metabolites from the gibberellin pathway. Plant J..

[36] Talon M., Koornneef M., Zeevaart J.A. (1990). Endogenous gibberellins in Arabidopsis thaliana and possible steps blocked in the biosynthetic pathways of the semidwarf ga4 and ga5 mutants. Proc. Natl. Acad. Sci. USA.

[37] Milborrow B.V. (2001). The pathway of biosynthesis of abscisic acid in vascular plants: A review of the present state of knowledge of ABA biosynthesis. J. Exp. Bot..

[38] French E., Iyer-Pascuzzi A.S. (2018). A role for the gibberellin pathway in biochar-mediated growth promotion. Sci. Rep..

[39] Macmillan J. (2001). Occurrence of Gibberellins in Vascular Plants, Fungi, and Bacteria. J. Plant Growth Regul..

[40] Yamaguchi S. (2008). Gibberellin Metabolism and its Regulation. Annu. Rev. Plant Biol..

[41] Csukasi F., Osorio S., Gutierrez J.R., Kitamura J., Giavalisco P., Nakajima M., Fernie A.R., Rathjen J.P., Botella M.A., Valpuesta V. (2011). Gibberellin biosynthesis and signalling during development of the strawberry receptacle. New Phytol..

[42] Wang X., Zhao M., Wu W., Korir N.K., Qian Y., Wang Z. (2017). Comparative transcriptome analysis of berry-sizing effects of gibberellin (GA3) on seedless *Vitis vinifera* L.. Genes Genom..

[43] Murcia G., Pontin M., Piccoli P. (2017). Role of ABA and Gibberellin A3 on gene expression pattern of sugar transporters and invertases in *Vitis vinifera* cv. Malbec during berry ripening. Plant Growth Regul..

[44] Fasoli M., Santo S.D., Zenoni S., Tornielli G.B., Farina L., Zamboni A., Porceddu A., Venturini L., Bicego M., Murino V. (2012). The Grapevine Expression Atlas Reveals a Deep Transcriptome Shift Driving the Entire Plant into a Maturation Program. Plant Cell.

[45] Xie Z., Su Z., Wang W., Guan L., Bai Y., Zhu X., Wang X., Jia H., Fang J., Wang C. (2019). Characterization of VvSPL18 and Its Expression in Response to Exogenous Hormones during Grape Berry Development and Ripening. Cytogenet. Genome Res..

[46] Moyano-Cañete E., Bellido M.L., García-Caparrós N., Medina-Puche L., Amil-Ruiz F., González-Reyes J.A., Caballero J.L., Garciía-Limones C., Blanco-Portales R. (2012). FaGAST2, a Strawberry Ripening-Related Gene, Acts Together with FaGAST1 to Determine Cell Size of the Fruit Receptacle. Plant Cell Physiol..

[47] An L., Ma J., Wang H., Li F., Qin D., Wu J., Zhu G., Zhang J., Yuan Y., Zhou L. (2018). NMR-based global metabolomics approach to decipher the metabolic effects of three plant growth regulators on strawberry maturation. Food Chem..

[48] Gu T., Jia S., Huang X., Wang L., Fu W., Huo G., Gan L., Ding J., Li Y. (2019). Transcriptome and hormone analyses provide insights into hormonal regulation in strawberry ripening. Planta.

[49] Kim J., Lee J.G., Hong Y., Lee E.J. (2019). Analysis of eight phytohormone concentrations, expression levels of ABA biosynthesis genes, and ripening-related transcription factors during fruit development in strawberry. J. Plant Physiol..

[50] Symons G.M., Chua Y.-J., Ross J.J., Quittenden L.J., Davies N.W., Reid J.B. (2012). Hormonal changes during non-climacteric ripening in strawberry. J. Exp. Bot..

[51] Martinez G.A., Chaves A.R., Añon M.C. (1994). Effect of gibberellic acid on ripening of strawberry fruits (*Fragaria annanassa* Duch.). J. Plant Growth Regul..

[52] Martinez G.A., Chaves A.R., Añon M.C. (1996). Effect of exogenous application of gibberellic acid on color change and phenylalanine ammonia-lyase, chlorophyllase, and peroxidase activities during ripening of strawberry fruit (*Fragaria* x *ananassa* Duch.). J. Plant Growth Regul..

[53] García-Luis A., Herrero-Villén A., Guardiola J. (1992). Effects of applications of gibberellic acid on late growth, maturation and pigmentation of the Clementine mandarin. Sci. Hortic..

[54] Alós E., Cercós M., Rodrigo M.J., Zacarías L., Talon M. (2006). Regulation of Color Break in Citrus Fruits. Changes in Pigment Profiling and Gene Expression Induced by Gibberellins and Nitrate, Two Ripening Retardants. J. Agric. Food Chem..

[55] Rodrigo M.J., Zacarias L. (2007). Effect of postharvest ethylene treatment on carotenoid accumulation and the expression of carotenoid biosynthetic genes in the flavedo of orange (*Citrus sinensis* L. Osbeck) fruit. Postharvest Biol. Technol..

[56] Gambetta G., Martínez-Fuentes A., Bentancur O., Mesejo C., Reig C., Gravina A., Agustí M. (2012). Hormonal and nutritional changes in the flavedo regulatingrind color development in sweet orange [*Citrus sinensis* (L.) Osb.]. J. Plant Growth Regul..

[57] Rodrigo M.J., Alquézar B., Alós E., Lado J., Zacarías L. (2013). Biochemical bases and molecular regulation of pigmentation in the peel of Citrus fruit. Sci. Hortic..

[58] Finkelstein R., Gibson S.I. (2002). ABA and sugar interactions regulating development: Cross-talk or voices in a crowd?. Curr. Opin. Plant Biol..

[59] Li Y., Lee K.K., Walsh S., Smith C., Hadingham S., Sorefan K., Cawley G.C., Bevan M. (2006). Establishing glucose- and ABA-regulated transcription networks in Arabidopsis by microarray analysis and promoter classification using a Relevance Vector Machine. Genome Res..

[60] Dekkers B.J.W., Schuurmans J.A.M.J., Smeekens S.C.M. (2008). Interaction between sugar and abscisic acid signalling during early seedling development in Arabidopsis. Plant Mol. Biol..

[61] Davies C., Boss P.K., Robinson S.P. (1997). Treatment of grape berries, a nonclimacteric fruit with a synthetic auxin, retards ripening and alters the expression of developmentally regulated genes. Plant Physiol..

[62] Deluc L.G., Quilici D.R., Decendit A., Grimplet J., Wheatley M.D., Schlauch K., Mérillon J.-M., Cushman J.C., Cramer G.R. (2009). Water deficit alters differentially metabolic pathways affecting important flavor and quality traits in grape berries of Cabernet Sauvignon and Chardonnay. BMC Genom..

[63] Mei Z., Ping L., Guanglian Z., Xiangxin L. (2009). Cloning and functional analysis of 9-cis-epoxycarotenoid dioxygenase (NCED) genes encoding a key enzyme during abscisic acid biosynthesis from peach and grape fruits. J. Plant Physiol..

[64] Gambettaa G.A., Matthews M.A., Shaghasi T.H., McElrone A.J., Castellarin S.D. (2010). Sugar and abscisic acid signaling orthologs are activated at the onset of ripening in grape. Planta.

[65] Çakir B., Agasse A., Gaillard C., Saumonneau A., Delrot S., Atanassova R. (2003). A Grape ASR Protein Involved in Sugar and Abscisic Acid Signaling. Plant Cell.

[66] Pourtau N., Mares M., Purdy S., Quentin N., Ruël A., Wingler A. (2004). Interactions of abscisic acid and sugar signaling in the regulation of leaf senescence. Planta.

[67] Hiratsuka S., Onodera H., Kawai Y., Kubo T., Itoh H., Wada R. (2001). ABA and sugar effects on anthocyanin formation in grape berry cultured in vitro. Sci. Hortic..

[68] Larronde F., Krisa S., Decendit A., Chèze C., Deffieux G., Mérillon J.-M. (1998). Regulation of polyphenol production in *Vitis vinifera* cell suspension cultures by sugars. Plant Cell Rep..

[69] Matsushima J., Hiratsuka S., Taniguchi N., Wada R., Suzaki N. (1989). Anthocyanin accumulation and sugar content in the skin of grape cultivar Olympia treated with ABA. J. Jpn. Soc. Hortic. Sci..

[70] Pirie A., Mullins M.G. (1976). Changes in Anthocyanin and Phenolics Content of Grapevine Leaf and Fruit Tissues Treated with Sucrose, Nitrate, and Abscisic Acid. Plant Physiol..

[71] Eveland A.L., Jackson D. (2012). Sugars, signalling, and plant development. J. Exp. Bot..

[72] Wu G.A., Terol J., Ibanez V., López-García A., Pérez-Román E., Borredá C., Domingo C., Tadeo F.R., Carbonell-Caballero J., Alonso R. (2018). Genomics of the origin and evolution of Citrus. Nat. Cell Biol..

[73] Alós E., Roca M., Iglesias D.J., Minguez-Mosquera M.I., Damasceno C.M., Thannhauser T.W., Rose J.K., Talon M., Cercos M. (2008). An evaluation of the basis and consequences of a stay-green mutation in the navel negra (nan) citrus mutant using transcriptomic and proteomic profiling and metabolite analysis. Plant Physiol..

[74] Peña L., Martín-Trillo M., Juárez J., Pina J.A., Navarro L., Martínez-Zapater J.M. (2001). Constitutive expression of Arabidopsis LEAFY or APETALA1 genes in citrus reduces their generation time. Nat. Biotechnol..

[75] Jia H., Orbovic V., Jones J.B., Wang N. (2016). Modification of the PthA4 effector binding elements in Type I CsLOB1 promoter using Cas9/sgRNA to produce transgenic Duncan grapefruit alleviating XccDpthA4:dCsLOB1.3 infection. Plant Biotechnol. J..

[76] Dutt M., Barthe G., Irey M., Grosser J. (2015). Transgenic Citrus Expressing an Arabidopsis NPR1 Gene Exhibit Enhanced Resistance against Huanglongbing (HLB.; Citrus Greening). PLoS ONE.

[77] Romero P., Rodrigo M.J., Alférez F., Ballester A.-R., González-Candelas L., Zacarías L., Lafuente M.T. (2012). Unravelling molecular responses to moderate dehydration in harvested fruit of sweet orange (*Citrus sinensis* L. Osbeck) using a fruit-specific ABA-deficient mutant. J. Exp. Bot..

[78] Romero P., Lafuente M.T., Alferez F. (2014). A transcriptional approach to unravel the connection between phospholipases A2 and D and ABA signal in citrus under water stress. Plant Physiol. Biochem..

[79] Rodrigo M.J., Alquézar B., Zacarías L. (2006). Cloning and characterization of two9-cis-epoxycarotenoid dioxygenase genes, differentially regulated during fruitmaturation and under stress conditions, from orange (*Citrus sinensis* L. Osbeck). J. Exp. Bot..

[80] Alferez F., Zacarias L., Kanellis A.K., Chang C., Klee H., Bleecker A.B., Pech J.C., Grierson D. (1999). Interaction between ethylene and abscisic acid in the regulation of citrus fruit maturation. Biology and Biotechnology of the Plant Hormone Ethylene II.

[81] Romero P., Gandía M., Alferez F. (2013). Interplay between ABA and phospholipases A2 and D in the response of citrus fruit to postharvest dehydration. Plant Physiol. Biochem..

